# Longitudinal Alterations in Prefrontal Resting Brain Connectivity in Non-Treatment-Seeking Young Adults With Cannabis Use Disorder

**DOI:** 10.3389/fpsyt.2019.00514

**Published:** 2019-07-25

**Authors:** Jazmin Camchong, Paul F. Collins, Mary P. Becker, Kelvin O. Lim, Monica Luciana

**Affiliations:** ^1^Department of Psychiatry, University of Minnesota, Minneapolis, MN, United States; ^2^Department of Psychology, University of Minnesota, Minneapolis, MN, United States; ^3^Department of Psychiatry, Hennepin Healthcare, Richfield, MN, United States

**Keywords:** cannabis, change, functional connectivity, longitudinal, anterior cingulate, non-treatment seeking

## Abstract

**Background:** Cannabis is increasingly perceived as a harmless drug by recreational users, yet chronic use may impact brain changes into adulthood. Repeated cannabis exposure has been associated with enduring synaptic changes in executive control and reward networks. It is important to determine whether there are brain functional alterations within these networks in individuals that do not seek treatment for chronic cannabis abuse.

**Methods:** This longitudinal study compared resting-state functional connectivity changes in executive control and reward networks between 23 non-treatment-seeking young adults with cannabis use disorder (6 females; baseline age M = 19.3 ± 1.18) and 21 age-matched controls (10 females; baseline age M = 19.4 ± 0.65) to determine group differences in the temporal trajectories of resting-state functional connectivity across a 2-year span.

**Results:** Results showed i) significant increases in resting-state functional connectivity between the caudal anterior cingulate cortex and precentral and parietal regions over time in the control group, but not in the cannabis use disorder group, and ii) sustained lower resting-state functional connectivity of anterior cingulate cortex seeds with frontal and thalamic regions in the cannabis use disorder group vs. the age-matched controls. Resting-state functional connectivity strength was correlated with cannabis use patterns in the cannabis use disorder sample.

**Conclusion:** Longitudinal alterations in intrinsic functional organization of executive control networks found in non-treatment-seeking young adults with cannabis use disorder (when compared to age-matched controls) may impact regulatory control over substance use behavior. Current findings were limited to examining executive control and reward networks seeded in ACC and NAcc, respectively. Future studies with larger sample sizes and enough power are needed to conduct exploratory analyses examining rsFC of other networks beyond those within the scope of the current study.

## Introduction

Cannabis is increasingly perceived as a harmless substance (1). Nearly 52% of 18- to 25-year olds in the USA report lifetime cannabis use (NIDA, 2014), with increasing rates of use among young adults. Many are chronic users who do not feel the need to stop using or seek treatment. Given these trends, it is important to investigate the impact of regular cannabis use on brain networks in young adults. Animal model evidence suggests that cannabis use may disrupt normative patterns of synaptic pruning (2). Chronic cannabis use alters synaptic pruning in the endocannabinoid system (3), which is involved in mediating executive function and reward processing (4), adversely affecting cognition and behavior (5–7). Thus, chronic cannabis use during critical developmental stages may affect aspects of integrity within networks that play a crucial role in drug-seeking behavior, such as the executive and reward processing networks, through enduring neurochemical alterations (8, 9).

The existing neuroimaging literature documents functional alterations in regulatory networks involved in executive functioning in heavy and frequent cannabis users using task-based functional magnetic resonance imaging (fMRI) (8, 10, 11). However, findings are inconsistent. For instance, *lower* prefrontal activity in the anterior cingulate cortex (ACC), relative to controls, has been associated with poor gambling task performance in cannabis use disorder (12). Conversely, *higher* prefrontal functional connectivity, relative to controls, in cannabis use disorder has been observed in the context of normal levels of executive control (13), suggesting a compensatory mechanism. Inconsistencies across studies may be related to individual differences in cognitive abilities, engagement, or fMRI-based task demands. To assist with this interpretation, it is essential to examine the integrity of executive functioning networks in cannabis use disorder without potential confounds associated with task-evoked brain activity.

The examination of synchronized patterns of signal fluctuation during rest (e.g., resting-state functional connectivity, rsFC) allows us to measure intrinsic neural network function and organization irrespective of task-based processes (14–16). RsFC-defined networks are reliably observed across studies and change with time in developmental populations (17, 18). For instance, Kelly et al. (17) assessed age-related shifts of multiple rsFC networks seeded in the ACC in a healthy sample without substance abuse and showed specific age-related differences between childhood and young adulthood in discrete executive control networks. Using a similar approach, our group previously reported specific rsFC ACC longitudinal alterations in treatment-seeking adolescents with cannabis use disorder (19). We reported that treatment-seeking adolescents with cannabis use disorder both lacked longitudinal increases in rsFC between the caudal ACC and superior frontal gyrus found in healthy controls, and showed longitudinal decreases in rsFC between the caudal ACC and orbitofrontal cortex. Moreover, we reported that reduced caudal ACC–orbitofrontal cortex rsFC at baseline was associated with higher cannabis consumption during the subsequent 18 months (19). Findings from our previous study were specific to adolescents who have been diagnosed with cannabis use disorder and had sought treatment. It is important to determine whether similar rsFC alterations exist in individuals with cannabis use disorder (CUD) who have not sought treatment and who vary in age.

While the above evidence suggests ACC alterations in CUD, reports have not been consistent in studies examining non-treatment-seeking individuals with CUD, with some studies reporting higher rsFC and task-based functional connectivity (FC). For example, a cross-sectional study that compared rsFC between non-treatment-seeking young adults with heavy cannabis use and controls (20) reported higher rsFC between the caudal ACC and medial frontal gyrus/precentral gyrus during rest in those with CUD versus controls. There is also cross-sectional evidence of specific task-based FC alterations in non-treatment-seeking individuals with CUD, characterized by higher ACC–amygdala FC and lower nucleus accumbens–orbitofrontal–hippocampus FC in those with CUD when compared to those without CUD (21). Inconsistency in these cross-sectional observations of altered ACC FC in non-treatment-seeking individuals may be related to different neuroimaging methodologies or different types of engagement during functional MRI scans (rest vs. task). Moreover, FC alterations may be different across time. The longitudinal changes in rsFC across time in non-treatment-seeking individuals with CUD, however, have not been reported in the literature.

Given that the prefrontal cortex is an important hub for executive control processes, much of the literature has examined and identified prefrontal circuitry alterations in CUD. Subcortical regions, however, are also implicated in drug addiction. The nucleus accumbens (NAcc) is crucially involved in processing the reinforcing effects of drugs (22). Repeated drug exposure generates long-lasting synaptic reorganization of NAcc’s cortical connections (23). Therefore, rsFC of the NAcc may be altered in CUD. A recent cross-sectional study reported altered frontal–striatal rsFC in non-treatment-seeking individuals with cannabis use disorder, with higher rsFC between a NAcc seed and rostral ACC and dorsomedial prefrontal cortex in those with cannabis use disorder vs. healthy controls (24). There is, however, no longitudinal evidence of altered changes in NAcc rsFC in non-treatment-seeking individuals with CUD.

The current study bridges gaps in the literature by comparing changes in rsFC of executive control (i.e., ACC) and reward networks (i.e., NAcc) across time in non-treatment-seeking young adults with CUD versus healthy controls. Because of similar resting scan parameters and similar neuroimaging methodologies, we expect to find similar alterations reported in our previous study of treatment-seeking adolescents with CUD (19).

In addition to identifying rsFC differences between CUD and healthy controls, it is important to determine how identified rsFC alterations are associated with substance use behaviors. Given that earlier ages of onset and higher frequencies and magnitudes of use have been associated with neural anomalies (25, 26), the current study specifically sought to determine the relationship between rsFC alterations and cannabis use characteristics.

The main study aim was to determine whether there are specific differences in executive control (i.e., ACC) and reward (NAcc) network rsFC changes across time between non-treatment-seeking young adults with cannabis use disorder (CUD) and healthy controls (HC). Because substance dependence affects rsFC (19, 27) and because cannabis impacts rsFC changes in adolescence (19), we expected to similar ACC and NAcc rsFC network alterations in young adults with CUD as those identified in our previous study (19). Specifically, based on previous findings in adolescents with cannabis use disorder (19), we hypothesized that young adults with CUD would show reduced changes in rsFC between the caudal ACC and frontal regions when compared to HC. Additionally, we hypothesized that individuals with CUD would show altered rsFC at each time point as well as altered rsFC changes between the NAcc and frontal regions relative to controls (24). Finally, based on previous reports of dose-related effects (26), and literature on the role of ACC in reward-based decision making and learning (28, 29), we hypothesized that observed altered rsFC would be correlated with cannabis use metrics. In order to maintain rigor and reproducibility, the scope of the current analysis focused on ACC and NAcc networks as an expansion of our previous findings in adolescents with CUD (19). Therefore, the direction of hypothesized effects could vary in other networks, and the current approach and subsequent findings should not be generalized to other brain networks.

## Materials and Methods

### Participants

Data from 44 participants (aged 18–21 years; **Table 1**) who provided informed consent under an IRB-approved University of Minnesota protocol were used in the current study. All subjects gave written informed consent in accordance with the Declaration of Helsinki. Participants included 23 college students who reported recreational cannabis use and received research diagnoses of cannabis use disorder (CUD, see below; 6 females) and 21 healthy controls (HC; 10 females).

**Table 1:** Demographics for non-treatment-seeking young adults with cannabis use disorder (CUD) and healthy controls (HC). Unless otherwise indicated, values represent means and, in parentheses, standard deviations. Statistics are presented for the main effect of group in repeated-measures ANOVAs, one-way ANOVAs, or chi-square analyses as appropriate. Significant group-by-time interactions are described below and in the text.

	Time 1		Time 2		Group main effect		Time main effect		Interaction effect	
	CUD (*N* = 23)	HC (*N* = 21)	CUD (*N* = 23)	HC (*N* = 21)	F/X^2^	p	F/X^2^	p	F/X^2^	p
Gender (% females)	28%	43.47%	–	–	*X* ^2^ = 2.12	.14	–	–	–	–
Mean age (SD)	19.4 (0.65)	19.3 (1.18)	21.8 (0.81)	21.5 (1.11)	*F* = 0.71	.40	917.08	.000	1.05	.312
Estimated IQ^1^	114.3 (10.5)	120.9 (8.5)	116.7 (8.2)	123.8 (8.7)	*F* = 6.06	.02	5.66	.022	.01	.943
Maternal education (years)^2^	16.2 (2.22)	16.1 (4.13)	–	–	*F* = 0.03	.86	–	–	–	–
Paternal education (years)	16.5 (2.11)	15.8 (4.58)	–	–	*F* = 0.42	.52	–	–	–	–
Mean number of years between MRI scans (SD)	–	–	2.35 (0.31)	2.20 (0.64)	1.04	.31	–	–	–	–
Nicotine (times used per day), past 6 months^3^	1.05 (1.72)	0.85 (3.36)	2.37 (3.18)	0.25 (0.53)	3.56	.06	0.58	0.45	4.10	.050
Alcohol use frequency,^4^ past 3 months (SD)	2.48 (0.73)	1.14 (1.01)	2.74 (0.92)	1.81 (0.98)	23.33	.000	11.08	.002	2.91	.095
Cannabis use frequency, past 3 months (SD)	4.13 (0.63)	0.48 (0.75)	3.65 (1.40)	0.62 (0.87)	184.55	.000	1.17	0.29	4.01	0.05

^1^Estimated IQ based on vocabulary and matrix reasoning scores from the Wechsler Abbreviated Scales of Intelligence.

^2^Maternal education data missing for one individual in the CUD group and one individual in the HC group.

^3^Modal daily nicotine use, as measured by a single-item self-report questionnaire, in HC = 0 (21 of 23 participants at baseline, 19 of 23 at FU). Groups were indistinct in nicotine use at study intake (F = .06, p = .81), but CUD used more at follow-up (F = 8.56, p = .006).

^4^PEI ratings: 0 = never; 1 = 1–5 times; 2 = 6–20 times; 3 = 21–49 times; 4 = 50–99 times; 5 = 100+ times. Group differences for nicotine, alcohol, and cannabis were supported by non-parametric statistics (Mann–Whitney U tests).

#### Recruitment and Inclusion/Exclusion Criteria

*CUD sample*. Interested participants, recruited from the University of Minnesota student population, responded to posted advertisements and completed a phone assessment of weekly and daily patterns of cannabis use as well as MRI eligibility.

*Inclusion criteria for the CUD* sample included self-reported near-daily cannabis use (the average was five times per week) for at least 1 year. Since cannabis use initiation is most common between ages 16 and 18 years in the United States (30), use onset was required to be before age 17. Potential CUD participants were excluded if they reported a history of psychological or medication treatment for emotional disorders. If basic eligibility criteria were met during the phone interview, an in-depth in-person clinical interview using the K-SADS (Kiddie Schedule for Affective Disorders and Schizophrenia) (31) followed, which assessed psychopathology by capturing lifetime histories of childhood disorders as well as current Axis-I psychopathology. CUDs had minimal psychopathology comorbidities: Two CUD participants met criteria for current or past bipolar disorder not otherwise specified (NOS). Two CUD met criteria for past oppositional defiant disorder and one for past specific phobia. The presence of these diagnoses did not impact reported findings, so all CUD participants were retained for analysis.

Substance use patterns were assessed with the K-SADS interview. CUDs were excluded if they reported daily cigarette use at the time of enrollment or if self-reported alcohol use exceeded four drinks (females) or five drinks (males) more than twice weekly. Recruited CUDs exhibited more symptoms related to problematic cannabis use than to problematic alcohol use. Individuals in the CUD sample met formal *DSM-IV* (*Diagnostic and Statistical Manual of Mental Disorders-IV*) criteria for cannabis dependence or abuse (*n* = 13 for cannabis use dependence, *n* = 9 for cannabis abuse). The average age of first cannabis use in the CUD sample was 15.2 years (SD = 1.2, range = 13 to 18 years).

Substance use frequencies were further queried with the self-reported Personal Experience Inventory (PEI) (32), which recorded the number of times participants used alcohol, cannabis, and other illicit substances within the last 3 months and 12 months and lifetime on a 5-point scale. An interview created on the basis of National Institute on Alcohol Abuse and Alcoholism guidelines was implemented to assess cannabis and alcohol use characteristics. Cannabis and alcohol use characteristics included: age of use onset (for cannabis and alcohol), frequency of use (for cannabis and alcohol), maximum number of cannabis hits/alcohol drinks in a 24-h period, and frequency of binge episodes (for cannabis and alcohol). These were quantified for the 30 days and 12 months prior to each assessment. Analyses for the current study focused on PEI-reported cannabis and alcohol use for the 3 months prior to each assessment (**Table 1**).

*HC sample*. Controls were drawn from a larger longitudinal study that began several years prior to the enrollment of the CUD sample. At initial enrollment into the larger study, controls were physically and psychologically healthy 9- to 23-year-olds (total *n* = 197) who responded to telephone solicitations, posted flyers, or direct mailings about the study. Those who were under the age of 18 at the study baseline were recruited through a metro community participant database maintained by the University of Minnesota’s Institute of Child Development. Those above the age of 18 were recruited through posted flyers throughout the community and direct mailings to non-academic staff at the University of Minnesota. At initial enrollment, controls were required to be physically healthy without a history of birth trauma, neurological illness, psychopathology, or learning disorders. Controls were excluded at initial enrollment if they met current or past *Diagnostic and Statistical Manual of Mental Disorders–Fourth Edition, Text Revision* (33) criteria for any psychiatric disorder as assessed with the K-SADS and/or if they reported regular recreational drug or alcohol use. At the controls’ baseline (Time 1) assessment, participants completed neurocognitive measures (34, 35), personality questionnaires, and a neuroimaging protocol. Control participants were tested every 2 years thereafter and repeated this battery (63). Resting-state MRI scans were not added to the study protocol until the second longitudinal assessment (Time 2). The CUD sample was recruited concurrently with the controls’ third longitudinal assessment (Time 3) and was administered the same battery of measures.

*Matching the CUD sample to HC*. Our goal was to match the groups by selecting controls from the same cohort as the CUD sample. All potential age-matched controls were identified from the larger study’s second and third assessments for matching to the CUD sample. Through this scheme, 58 potential controls at either control study Time 2 or control study Time 3 were identified. Potential controls were excluded from the current analysis if a) they did not complete the relevant Time 2-to-Time 3 or Time 3-to-Time 4 longitudinal assessments (*n* = 23), b) they were not scanned at control study Time 3 due to either budgetary limitations (*n* = 9)^1^ or scanning contraindications (e.g., pregnancy, metal implants) (*n* = 2), or c) their resting-state imaging data were not usable (*n* = 3). This approach yielded the 21 HCs that are included in this analysis. While HCs did report some alcohol use, CUD and HC differed in the extent of alcohol use, with higher use in CUDs. Alcohol use was used as a covariate in the analyses. Groups were statistically similar in age, gender distribution, parental education, and initial recent nicotine use, as indicated in **Table 1**. While there was some nicotine use reported in the recent past, no participants reported that they were currently (at the time of assessment) daily smokers.

*Inclusions for all participants (CUD and HC)* included being a native English speaker; right-handed as assessed by the Edinburgh Inventory (36); with normal/corrected-to-normal vision and hearing; and without a history of neurological problems, intellectual impairment, birth complications, chronic illness, or current pregnancy.

All participants were asked to refrain from alcohol and drug use for 24 h before testing. Because the study’s primary goal was to examine rsFC changes across time in the context of active cannabis use and because long-term abstinence was not required, formal drug testing was not implemented.

### Imaging Acquisition

Participants were tested twice with a 2-year span between assessments. At both assessments, participants underwent a 6-min resting-state fMRI scan. Imaging occurred at the same time of day (mornings) for all participants. Interscan interval was equivalent between groups (**Table 1**). Images were collected using a Siemens TIM Trio 3T scanner (Erlangen, Germany). Sequence parameters were as follows: gradient-echo echo-planar imaging (EPI) 180 volumes, repetition time (TR) = 2 s, echo time (TE) = 30 ms, flip angle = 90°, 34 contiguous anterior commissure and posterior commissure aligned axial slices with an interleaved acquisition, voxel size = 3.4 × 3.4 × 4.0 mm, matrix = 64 × 64 × 34. Three-dimensional structural brain images were obtained with a coronal T_1_-weighted magnetization-prepared rapid gradient echo (MPRAGE) sequence (TR = 2,530 ms, TE = 3.65 ms, time interval = 1,100 ms, 240 slices, voxel size = 1.0 × 1.0 × 1.0, flip angle = 7°, field of view = 256 mm). A field map acquisition was used to correct the fMRI data for geometric distortion caused by magnetic field inhomogeneities (TR = 300 ms, TE = 1.91 ms/4.37 ms, flip angle = 55°, voxel size = 3.4 × 3.4 × 4.0 mm).

During the resting-state scan, participants were instructed to close their eyes, to remain still, and to remain awake. At the end of the scan, participants were asked if they remained awake. All participants were communicative between scanning sequences, and no participants reported falling asleep. Because scan duration of the current study was short (6 min), it is likely that participant impressions are accurate (37).

### Imaging Analysis

#### Individual-Level Analyses

##### Data Preprocessing

Imaging data were preprocessed using Analysis of Functional NeuroImages (AFNI) and FMRIB Software Libraries (FSL; Oxford, United Kingdom), similar to our previous studies (19, 27, 38). Preprocessing consisted of: dropping the first three volumes to account for magnetic field homogenization; B0 field map unwarping, slice time correction; three-dimensional motion correction (AFNI: 3dvolreg^2^ was used to register each 3-D volume to a base volume to correct for motion); skull stripping; spatial smoothing (with a 6 mm full-width half-maximum kernel); and grand mean scaling, high-pass temporal filtering (.01 Hz) to remove low-frequency drift, and registration of all images to Montreal Neurological Institute (MNI) 2 × 2 × 2 mm standard space.

Motion correction was conducted in two steps (19, 27, 38). First, three-dimensional motion correction calculation provided motion correction parameters for each participant (AFNI: 3dvolreg). CUD and HC groups did not statistically differ along the six motion parameters (*p* > .05, 2-tailed), corresponding to framewise displacement (translation in millimeters) and rotation (in degrees; roll, pitch, yaw) along *x*−, *y*−, and *z*− dimensions, at either at the first (**Table 2**) or subsequent longitudinal (**Table 3**) rest fMRI sessions. Second, motion-corrected individual preprocessed data were then denoised, a method that included selection and regression of independent components corresponding to head motion (see **Section Data Denoising** next for description).

**Table 2:** Summary movement parameters at baseline after three-dimensional motion correction (AFNI: 3dvolreg) showing rotation (in degrees; roll, pitch, yaw) and translation (in millimeters; superior/inferior, left/right, posterior/anterior) motion parameters.

	Group	N	Mean	Standard deviation	t	Degrees of freedom	Significance (2-tailed)
Roll	HC	21	−.00040	.00180			
	CUD	23	.00070	.00439	−1.109	29.736	.276
Pitch	HC	21	.00049	.00331			
	CUD	23	−.00087	.01223	.517	25.499	.610
Yaw	HC	21	−.00025	.00130			
	CUD	23	−.00046	.00138	.519	41.955	.607
Superior/Inferior	HC	21	.00004	.00420			
	CUD	23	−.00030	.00068	.382	20.975	.706
Left/Right	HC	21	−.00022	.00100			
	CUD	23	.00033	.00238	−1.038	30.123	.307
Posterior/ Anterior	HC	21	.00026	.00051			
	CUD	23	.00123	.00641	−.723	22.316	.477

**Table 3:** Summary movement parameters at follow-up after three-dimensional motion correction (AFNI: 3dvolreg) showing rotation (in degrees; roll, pitch, yaw) and translation (in millimeters; superior/inferior, left/right, posterior/anterior) motion parameters.

	Group	N	Mean	Standard deviation	t	Degrees of freedom	Significance (2-tailed)
Roll	HC	21	−.00045	.00122			
	CUD	23	−.00022	.00045	−.811	24.895	.425
Pitch	HC	21	.00015	.00067			
	CUD	23	−.000069	.00088	.901	40.780	.373
Yaw	HC	21	−.00017	.00077			
	CUD	23	−.00007	.00049	−.495	33.336	.624
Superior/Inferior	HC	21	−.00004	.00084			
	CUD	23	−.00001	.00065	−.172	37.626	.864
Left/Right	HC	21	−.00026	.00049			
	CUD	23	.00001	.00031	−2.176	33.761	.037
Posterior/ Anterior	HC	21	.00002	.00045			
	CUD	23	.00001	.00035	.129	37.879	.898

##### Data Denoising

Independent component analysis [FSL, MELODIC (Multivariate Exploratory Linear Optimized Decomposition into Independent Components)] was used to decompose individual preprocessed 4-D data sets into different spatial and temporal components.^3^ Dimensionality of the individual components analysis reduction was automatic. Resulting independent components were classified as noise using spatial and temporal characteristics detailed in the MELODIC manual^4^ and were based on previous methodological reports that describe identification of individual sources of artifact (17, 39). Independent components were identified as representing noise corresponding to head motion (i.e., “rim-like” artifacts around the brain, spikes in time series), scanner artifacts (i.e., slice dropouts, high-frequency noise, field inhomogeneities), and physiological noise (i.e., respiration, cardiac frequencies, white matter signal, ventricular/cerebrospinal fluid fluctuations, frontal air cavities, ocular structures) with a customized in-house program written in Python. Signals from noise components were regressed from the preprocessed data (FSL: fsl_regfilt). All independent components representing major sources of artifacts were removed while preserving the integrity of the continuous time series.

To determine whether the variance in fMRI signal in the current data varied systematically across time or varied systematically by group, a repeated-measures ANOVA was conducted [as in Refs. (40, 41)] defining the sum of total percent variance accounted for by components removed (noise) as the dependent variable, the effect of time (baseline vs. follow-up) as the within-subject factor, and the effect of group (CUD vs. HC) as the between-subjects factor. Data showed: i) no main effect of time, *F*(1,42) = 0.656, *p* = 0.422, suggesting that noise was stable across time; ii) no main effect of group, *F*(1,42) = 2.243, *p* = 0.142, suggesting that noise was not significantly different between CUD and HC; and iii) no significant group × time interaction, *F*(1,42) = 2.099, *p* = 0.155, suggesting that the amount of percent variance accounted for by noise did not change differentially across time depending on the group. Analyses of covariance (ANCOVA) controlling for this measure (total percent variance accounted for by components removed) were conducted to investigate whether differences in percent variance due to noise affected rsFC results (see sections Interaction Effects: Differences in rsFC Changes between Groups and Sustained Group rsFC Differences).

#### Region of Interest (ROI) Selection and Seed Generation

**Figure 1:**
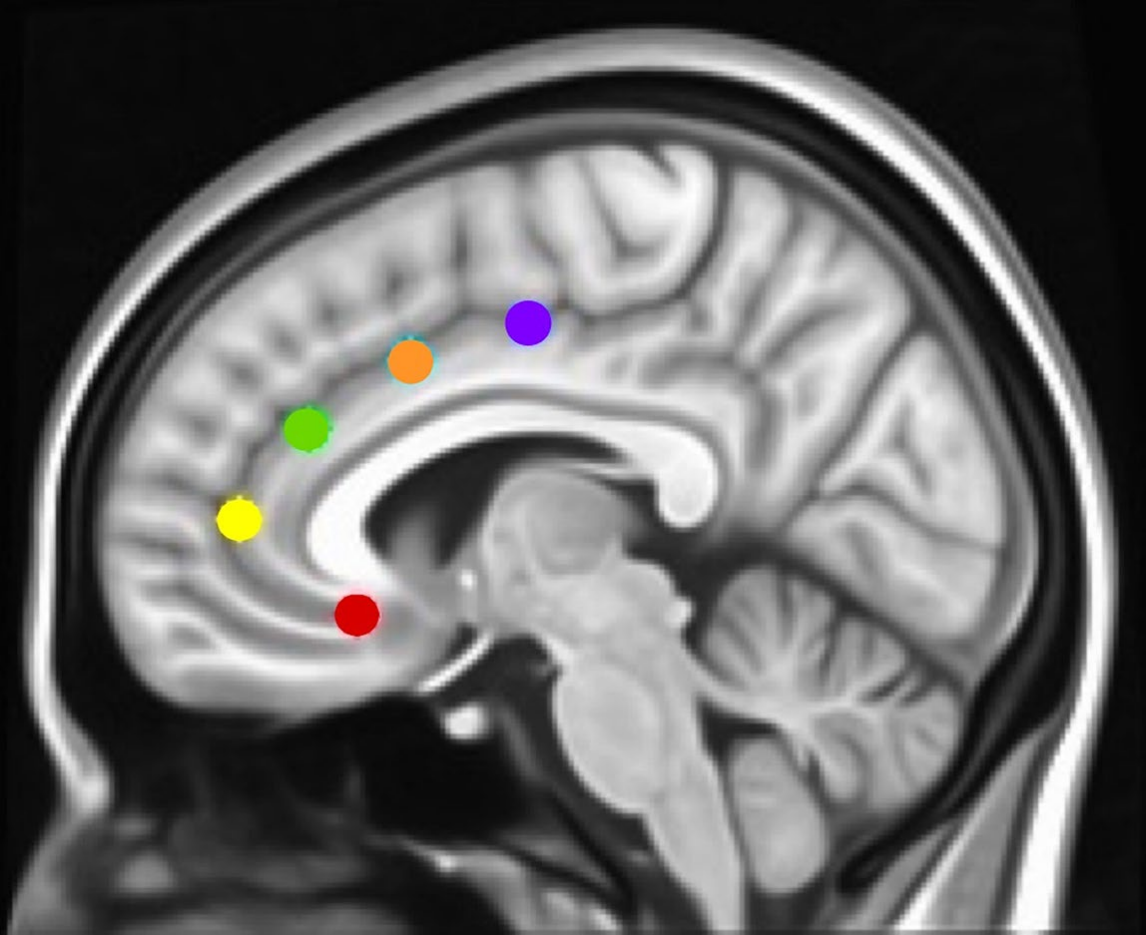
Montreal Neurological Institute (MNI) sagittal view showing anterior cingulate cortex (ACC) seeds. Using the same methods as in Margulies et al. and Kelly et al. (17, 42), and our studies [Camchong et al., e.g., Refs. (27, 45, 46)], we examined the rsFC (resting-state functional connectivity) of five bilateral seed regions of interest (ROIs) located along the ACC: caudal ACC (blue; MNI coordinates: *x* = ±5, *y* = 10, *z* = 47), dorsal ACC (cyan; *x* = ±5, *y* = 14, *z* = 42), rostral ACC (green; *x* = ±5, *y* = 34, *z* = 28), perigenual ACC (yellow; *x* = ±5, *y* = 47, *z* = 11), and subgenual ACC (red; *x* = ±5, *y* = 25, *z* = 10). Each spherical seed covered 257 voxels in 1 × 1 × 1mm space with a radius of 3.5 mm with left and right hemispheres combined.

To determine how networks associated with specific executive control domains changed across time between groups, we examined rsFC of five functionally distinct anterior cingulate cortex (ACC, **Figure 1**) networks for which changes across time have been examined in healthy controls as well as in adolescents with CUD (17, 19): caudal ACC [MNI (Montreal Neurological Institute) coordinates: *x* = ±5, *y* = 10, *z* = 47), dorsal ACC (*x* = ±5, *y* = 14, *z* = 42), rostral ACC (*x* = ±5, *y* = 34, *z* = 28), perigenual ACC (*x* = ±5, *y* = 47, *z* = 11), and subgenual ACC (*x* = ±5, *y* = 25, *z* = 10). Each of these ACC regions has been associated with specific executive control domains: motor control, cognitive/attentional control, conflict monitoring, internalization/mentalizing, and emotional regulation, respectively (17, 42). As a secondary analysis and to determine how reward processing networks might vary between groups, we examined rsFC of the nucleus accumbens [NAcc; MNI coordinates: *x* = 12, *y* = 10, *z* = −8 (right) and *x* = −10, *y* = 10, *z* = −8 (left)] (43), using 3.5 mm spherical seeds placed bilaterally [as in Ref. (27)]. Because we did not hypothesize laterality effects within ACC or NAcc networks (42, 44), and to minimize the number of regions of interest examined, we converged left and right seeds, resulting in five bilateral ACC seeds (17, 19) and one bilateral NAcc seed (27). Each bilateral spherical seed covered 257 voxels in 1 × 1 × 1 mm MNI space. For each participant’s preprocessed and denoised residual data, mean time series were extracted for each of the six bilateral seed regions.

#### Resting-State Individual-Level Functional Connectivity Analysis

**Figure 2:**
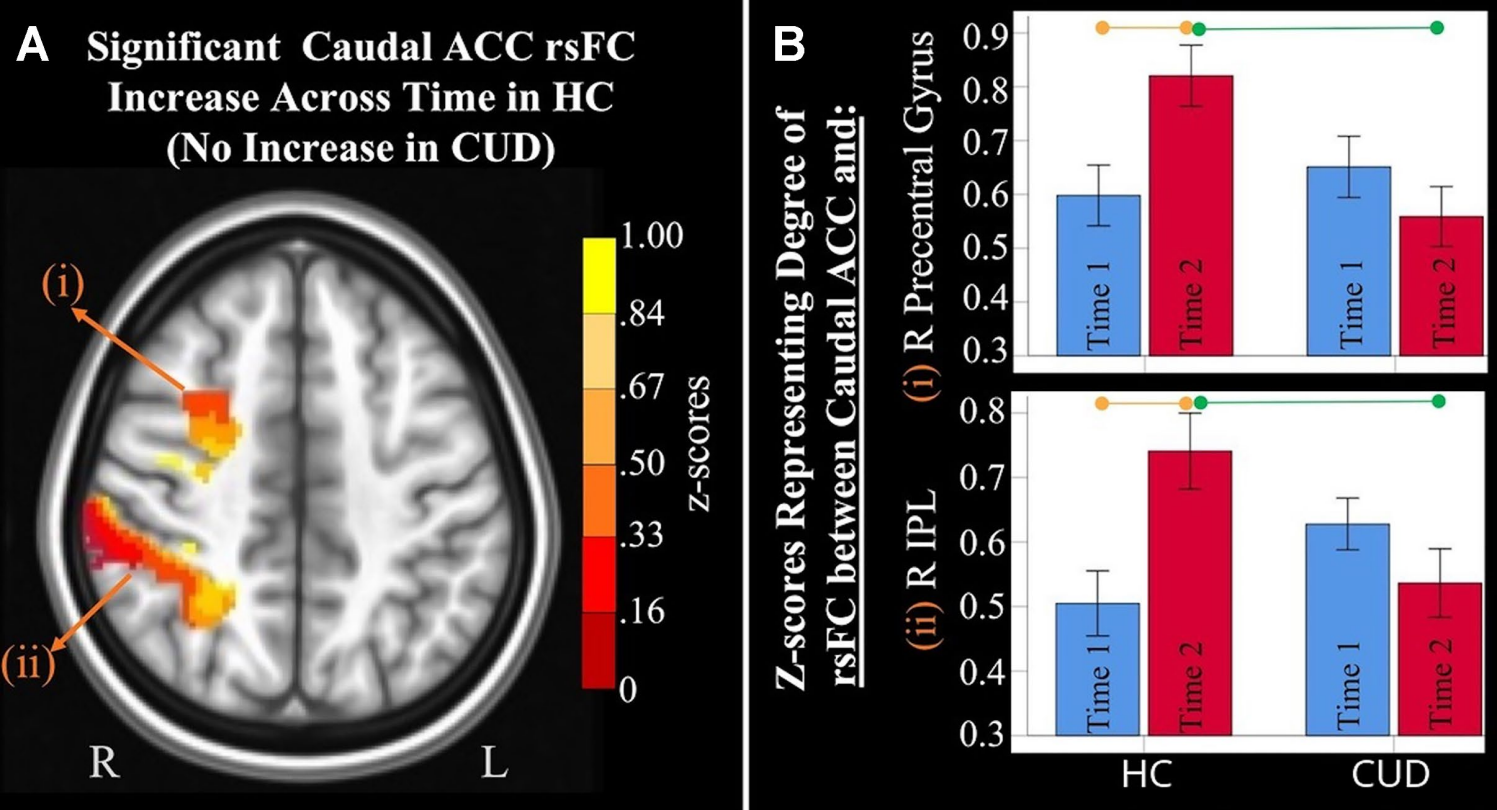
*Significant interaction effects* of caudal anterior cingulate cortex (ACC) resting-state functional connectivity (rsFC) identified by a 2 × 2 mixed-model analysis controlling for alcohol and nicotine. **(A)** LEFT: Axial MNI (Montreal Neurological Institute) brain slice (*z* = 46) displaying two clusters that showed a significant group × time interaction in resting-state functional connectivity (rsFC) between the caudal anterior cingulate cortex (ACC) seed and: (i) right (R) precentral gyrus (*F* = 9.467, *p* = 0.004; Brodmann area 6; 471 voxels) and (ii) R inferior parietal lobule (*F* = 8.348, *p* = 0.006; Brodmann area 7; 1,900 voxels). **(B)** RIGHT: Orange lines above HC bar graphs show significant *post hoc* comparisons in which healthy controls (HC) had significant rsFC increases from Time 1 (blue bars) to Time 2 (red bars) between caudal ACC and (i) right precentral gyrus (*F* = 9.983, *p* = 0.006) and (ii) right IPL (*F* = 13.505, *p* = 0.002). Individuals with cannabis use disorder (CUD), however, did not show any change in rsFC across time between these regions. Green lines between red bars show significantly lower rsFC in CUD than HC at Time 2 between caudal ACC and (i) right precentral gyrus (*F* = 4.992, *p* = 0.031) and (ii) right inferior parietal lobule (*F* = 4.568, *p* = 0.039).

For both time points, the average time series was extracted for each seed for each participant and averaged across hemispheres (AFNI: 3dROIstats^5^ was used to extract the mean signal fluctuation over time within each seed, resulting in an average waveform for each seed). Correlation analyses on the denoised data were performed between the extracted average time series from each bilateral seed and all brain voxels. Analyses were conducted for each seed separately (AFNI: 3dfim+^6^ was used to calculate the cross-correlation between the average waveform and the signal for each voxel in the brain). This generated maps with correlation coefficients (*r*) for each voxel, for each individual, for each bilateral seed region, at each time point. Correlation coefficients were transformed to Fisher’s *z* values (AFNI: 3dcalc^7^ was used to conduct voxel-by-voxel arithmetic calculations for the transformation). Values shown in **Figures 2** **–** **4** represent standardized *z* values.

**Figure 3:**
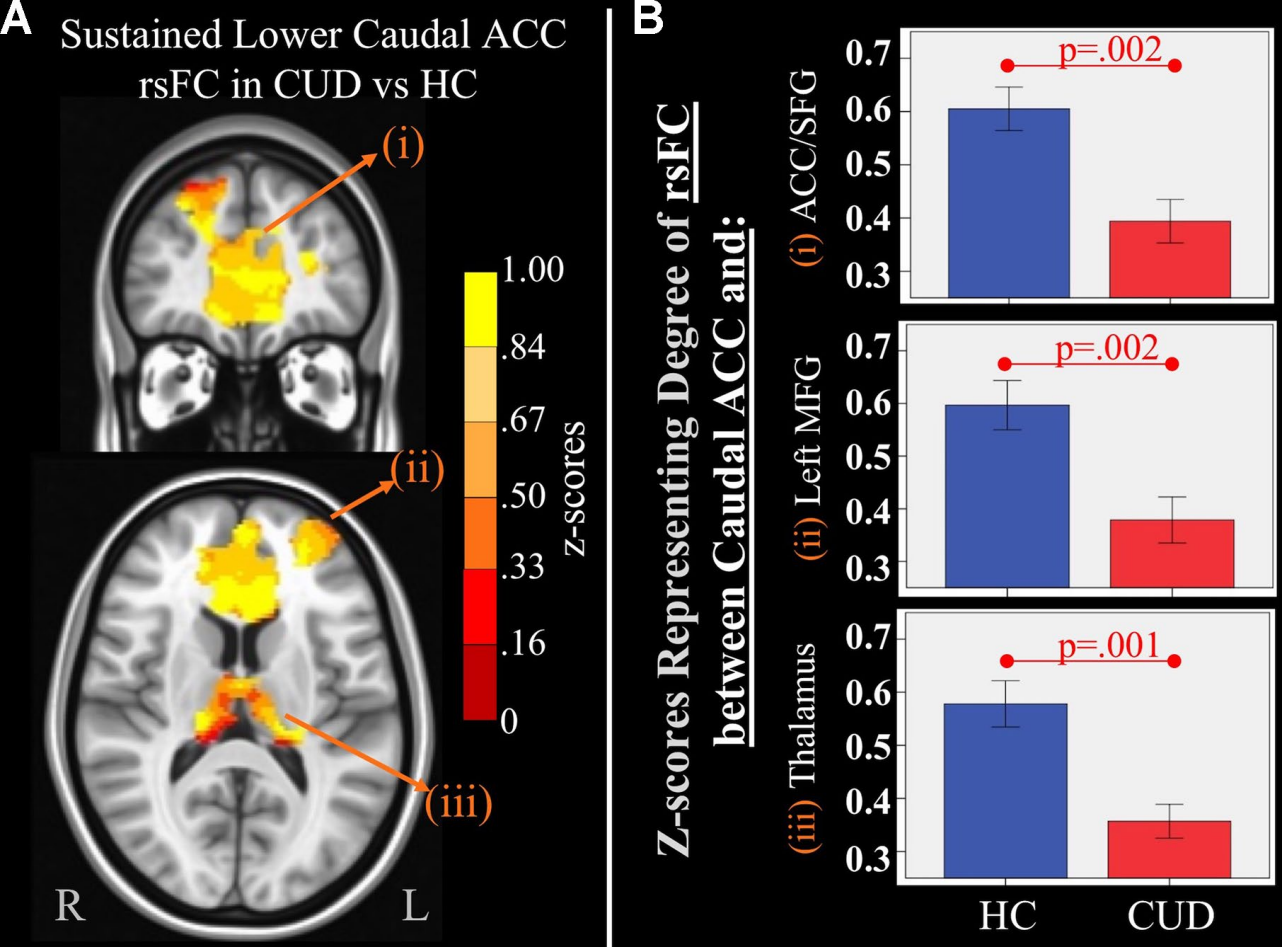
*Main effect of group* of caudal anterior cingulate cortex (ACC) resting-state functional connectivity (rsFC) identified by a 2 × 2 mixed-model (group × time) analysis controlling for alcohol and nicotine. **(A)** TOP LEFT: Coronal Montreal Neurological Institute (MNI) brain slice (*y* = 44) illustrating a cluster that showed a significant main effect of group in rsFC between the caudal ACC seed and (i) a cluster comprised of dorsal ACC, rostral ACC, and right superior frontal gyrus (ACC/SFG) (Brodmann areas 9, 10, and 32; 6,877 voxels). BOTTOM LEFT: Axial slice (*z* = 12) of MNI brain illustrating clusters that showed a significant main effect of group in rsFC between the caudal ACC seed and (ii) left (L) medial frontal gyrus (MFG) (Brodmann area 10; 684 voxels) and (iii) bilateral medial dorsal nucleus of the thalamus (1,940 voxels). **(B)** RIGHT: Bar graphs representing mean (error bars: +/− 1 standard error) illustrating significant main effects characterized by lower rsFC between caudal ACC and (i) ACC/SFG (*F* = 11.260, *p* = 0.002), (ii) left MFG (*F* = 10.630, *p* = 0.002, and (iii) medial dorsal nucleus of the thalamus (*F* = 11.888, *p* = 0.001) in individuals with cannabis use disorder (CUD) than healthy controls (HC) across time.

**Figure 4:**
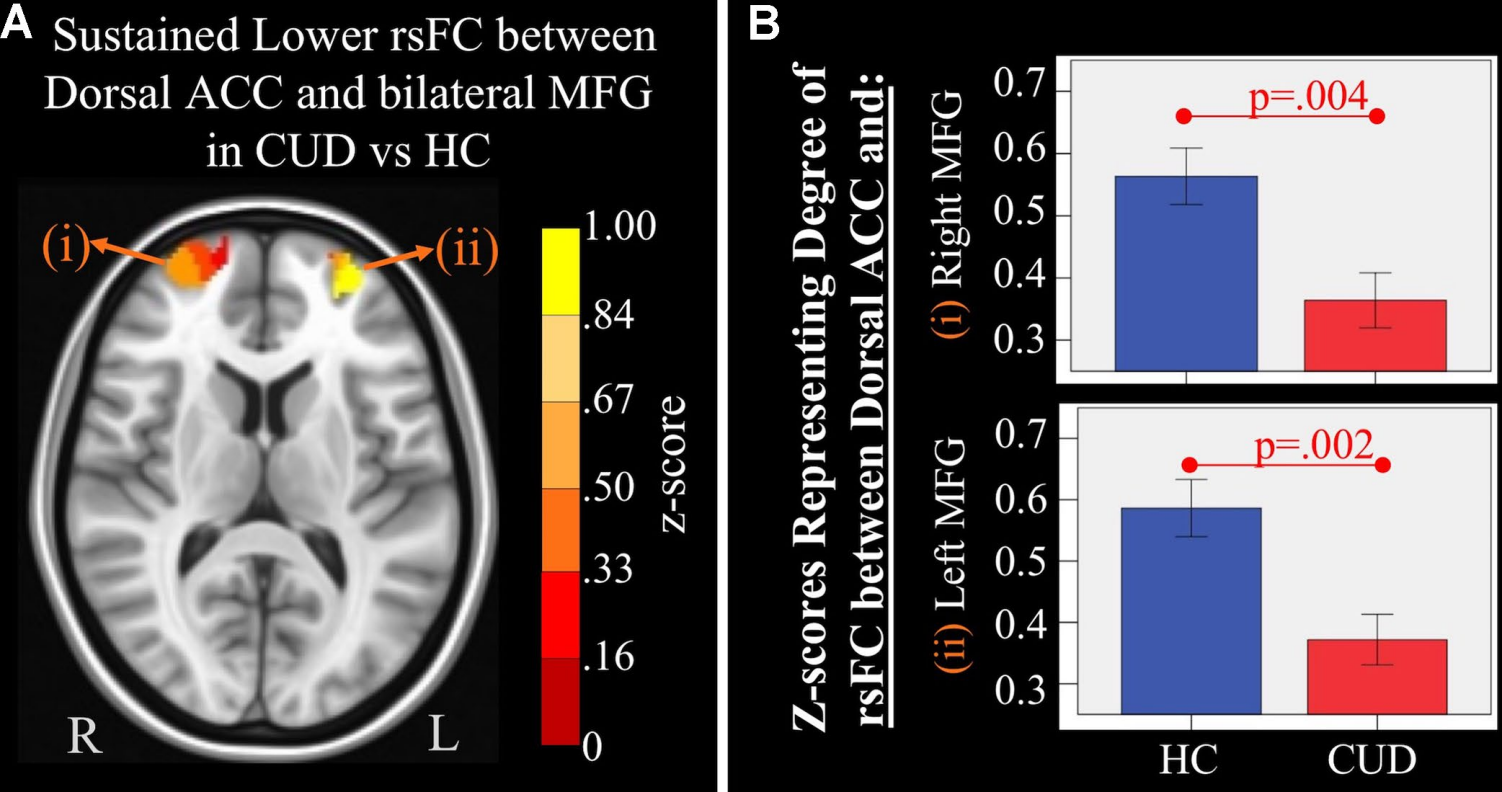
*Main effect of group* of dorsal anterior cingulate cortex (ACC) resting-state functional connectivity (rsFC) identified by a 2 × 2 mixed-model (group × time) analysis controlling for alcohol and nicotine. **(A)** LEFT: Axial Montreal Neurological Institute (MNI) brain slice (*z* = 11) illustrating clusters that showed a significant main effect of group in rsFC between the dorsal ACC seed and (i) right medial frontal gyrus (MFG; Brodmann area 10; 276 voxels) and (ii) left MFG (Brodmann area 10; 293 voxels). **(B)** RIGHT: Bar graphs representing mean (error bars: +/− 1 standard error) illustrating significant main effects characterized by lower rsFC between dorsal ACC and (i) right middle frontal gyrus (MFG) (*F* = 9.621, *p* = 0.004) and (ii) left MFG (*F* = 11.278, *p* = 0.002) in individuals with cannabis use disorder (CUD) than healthy controls (HC) across time.

#### Group-Level Analyses

Whole-brain analyses were conducted to investigate group differences in rsFC across time and to identify persistent group differences. Alcohol use (past 3 months’ alcohol use PEI rating, averaged across time points, **Table 1**) was covaried. Potential influences of nicotine and IQ on group differences were investigated *post hoc* as indicated in the sections Exploratory Analysis Investigating the Association Between rsFC and cannabis use and Exploratory Analysis Investigating the Association Between rsFC and Intelligence. To investigate whether CUD and HC had different patterns of rsFC change across time, we conducted mixed-effects analyses of covariance (ANCOVA; whole-brain analysis) using AFNI (3dLME^8^, a group-analysis program that performs linear mixed-effects modeling analysis) to assess main effects of group (CUD vs. HC), main effects of time (initial resting-state assessment vs. two-year follow-up), and group × time interaction effects.

##### Correction for Multiple Comparisons

Clustering and thresholding (47) were applied to maps resulting from ANCOVA analyses for each seed. To account for a non-Gaussian distribution of spatial auto-correlations within fMRI data (47), values representing smoothness of data on each resulting group statistics map were calculated (AFNI: 3dFWHMx^9^ was used to compute the full-width half-max or noise smoothness values for all volumes in each data set while taking into account a non-Gaussian distribution). The smoothness values were input into the 3dClustSim (AFNI: 3dClustSim^10^ was used to compute a cluster size threshold for a given voxel-wise *p*-value threshold) to estimate the probability of false-positive (noise-only) clusters for a voxel-wise *p*-value threshold of.001. For the caudal ACC seed at a per-voxel *p*-value threshold of 0.001, 3dClustSim estimated a cluster size threshold of 355 voxels to maintain a cluster-wise corrected *p*-value of .05. This cluster size was then used to threshold (*p* = 0.025, two-tailed 0.05) and cluster (AFNI: 3dmerge^11^ was used to edit the data set with specific thresholding and clustering limits) the statistics map for the caudal ACC seed (resulting from section Region of Interest (ROI) Selection and Seed Generation) with a connection distance of 5.6 (maximum number of grid cells apart voxels can be to be considered directly connected) and cluster volumes of at least 2,840 mm^3^ (355 voxels × 2 mm × 2 mm × 2 mm = 2,840 mm^3^ minimum cluster volume). Using the same parameters, minimum cluster size was: > 181 voxels for the dorsal ACC (1,448 mm^3^ minimum cluster volume), >248 voxels for the perigenual ACC (1,984 mm^3^ minimum cluster volume), >265 voxels for the rostral ACC (2,120 mm^3^ minimum cluster volume), >243 voxels for the subgenual ACC (1,944 mm^3^ minimum cluster volume), and >210 voxels for the NAcc (1,680 mm^3^ minimum cluster volume). Regions that survived clustering and thresholding were identified and used as masks from which individual *z*-scores were extracted for presentation and for exploration of the correlation between rsFC and cannabis use.

##### Exploratory Analysis Investigating the Association Between rsFC and Cannabis Use

Averaged *z*-scores from clusters that survived clustering and thresholding were used as dependent variables in regression models to determine rsFC correlations with frequencies and amounts of cannabis in the CUD sample, covarying for comorbid alcohol and nicotine use.

##### Exploratory Analysis Investigating the Association Between rsFC and Intelligence

Because controls demonstrated higher IQ scores than cannabis users (**Table 1**), we examined the influence of IQ on our reported findings in two ways. First, we examined the associations between rsFC values and IQ in the full sample with Pearson correlations. Next, we examined IQ’s association with the extracted connectivity data by adding it as a covariate to *post hoc* repeated-measures ANOVAs. That is, we assessed whether group and group × time differences in rsFC remained significant when controlling for IQ, alcohol, and nicotine use.

## Results

### Group Comparison

Group-level analyses revealed the following significant interaction and group-based main effects.

#### Interaction Effects: Differences in rsFC Changes Between Groups

Mixed-effects ANCOVA (controlling for alcohol use) revealed a significant group × time interaction in rsFC between caudal ACC and a) the right precentral gyrus and b) right inferior parietal lobule (**Figure 2**). Post hoc analyses revealed that only the HC group (not the CUD group) showed a significant increase in rsFC between these regions across time. There were no other ACC or NAcc regions that showed group × time interaction effects. These effects remained significant when alcohol use, nicotine use, and IQ were covaried.

Additionally, ANCOVA results (“Methods,” section Data Denoising) showed that after controlling for the effects of the sum of the percent of variance accounted for by noise, differences in all clusters, shown in **Figure 2**, were still significant (see *F*- and *P*-values in **Table 4**).

**Table 4:** *Significant group* **×* time interaction.* Montreal Neurological Institute (MNI) coordinates of clusters (center of mass) in which resting functional connectivity (rsFC) of caudal anterior cingulate cortex (ACC) showed a significant (*per-voxel p = .001, cluster p = 0.025, two-tailed p = 0.0* *5*) Group (non-treatment-seeking individuals with cannabis use disorder vs. healthy controls) by time (Time 1 vs. 2) interaction (**Figure 1**). No other significant interactions were found for other regions of interest. *F* and significance (Sig) *p*-values show analyses of covariance (ANCOVA) results controlling for the effects of differences in the sum of percent of variance accounted for by noise.

RsFC between Caudal ACC and:	Hemisphere	Brodmann area	*x*, *y*, *z*	F	Sig (*p*-value)	Cluster Size (# voxels)
Precentral gyrus	Right	6	30, −3, 50	14.09	.001	471
Inferior parietal lobule and precuneus	Right	7	40, −42, 48	12.46	.001	1,900

#### Sustained Group rsFC Differences

There were several significant main effects of group. CUD showed sustained lower rsFC (vs. HC) between the *caudal ACC seed* and: a) a cluster comprised of the right superior frontal gyrus, dorsal and rostral ACC (**Figures 3A-i and B-i**), b) left middle frontal gyrus (**Figures 3A-ii and B-ii**), and c) bilateral medial dorsal and anterior thalamic nuclei (**Figures 3A-iii and B-iii**). CUD also showed sustained lower rsFC (vs. HC) between the *dorsal ACC seed* and bilateral middle frontal gyrus (**Figure 4**). All but one effect (the group difference in dorsal ACC rsFC with the left middle frontal gyrus) remained significant when nicotine use and IQ, together with alcohol use, were also covaried in a *post hoc* analysis. Additionally, ANCOVA results (“Methods,” section Data Denoising) showed that after controlling for the effects of the sum of percent of variance accounted for by noise, differences in all clusters, shown in **Figures 3** and **4**, were still significant (see *F*- and *P*-values in **Table 5**).

**Table 5:** *Significant main effect of group*. Montreal Neurological Institute (MNI) coordinates of clusters (center of mass) in which resting functional connectivity (rsFC) of regions of interest (ROIs) showed a significant (per-voxel *p* = .001, cluster *p* = 0.025) main effect of group (non-treatment-seeking individuals with cannabis use disorder vs. healthy controls). No significant main effects of group were found for other regions of interest. *F* and significance (Sig) *p*-values show ANCOVA results controlling for the effects of differences in the sum of percent of variance accounted for by noise.

ROI	rsFC with:	Hemis-phere	Brod- mann area	*x*, *y*, *z*	F	Sig (*p*-value)	Cluster size (# voxels)
Caudal anterior cingulate cortex (ACC)	Medial dorsal nucleus of the thalamus	Bilateral	—	1, −18, 5	16.27	.00023	1,940
	Dorsal and rostral ACC and superior frontal gyrus (SFG)	Bilateral ACC, left SFG	9, 10, 32	−1, 39, 16	14.40	.00048	6,877
	Medial frontal gyrus	Left	10	−30, 54, 16	14.13	.00053	684
							
Dorsal ACC	Medial frontal gyrus and superior frontal gyrus	Left Right	10 10	−29, 59, 11 26, 61, 14	14.17 11.57	.00052 .00151	293 276

### Exploratory Analyses: Correlations Between Cannabis Use Metrics and rsFC

Associations between cannabis use metrics for the year prior to assessment and rsFC strength were examined within the CUD sample controlling for comorbid alcohol and nicotine use. Metrics included age of onset of cannabis use; reported frequency of use for the prior 30 days, prior 12 months, and lifetime; reported magnitude of use (e.g., number of hits) for the same time intervals; and the maximum number of hits in a 24-h period for the prior 30 days, prior 12 months, and lifetime. A higher number of binge occurrences, averaged for the year prior to each time point, was marginally associated with lower averaged rsFC between the dorsal ACC and right middle frontal gyrus (partial *r* = −.40, *p* < .10). Binge episodes were defined by asking participants to report the largest number of hits (deep inhales) consumed in a 24-h period and then to indicate the frequency of use at this level over the past year prior to assessment (e.g., by asking about the largest number of hits consumed in a 24-h period and the frequency of engaging in that behavior). Higher numbers of binge occurrences were also nominally associated with lower rsFC in all other connections that showed main effects of group (partial *r*’s ranged from −.28 to −.36), but these did not reach statistical significance, likely due to the small sample size. An earlier age of CUD onset was associated with *greater* rsFC between the dorsal ACC and right middle frontal gyrus (partial *r* = −.46, *p* < .05).

### Exploratory Analyses: Correlations Between rsFC and Intelligence

When examining correlations with both groups combined (HC and CUD), IQ was significantly positively associated with caudal ACC–right precentral gyrus rsFC at Time 1 (*r* = .38, *p* < .05) and at Time 2 (*r* = .34, *p* < .05). In the CUD sample alone, IQ was significantly and positively associated with rsFC between the caudal ACC and right inferior parietal lobule at Time 2 (*r* = .44, *p* < .04) but not with any of the other regions that showed group differences. No significant IQ correlates were found in the controls.

When adding IQ as a covariate to *post hoc* repeated-measures ANOVAs (controlling for IQ, alcohol use, and nicotine use), the group x time effects for a) caudal ACC to the right precentral region and b) caudal ACC to the right inferior parietal region remained significant (*p* < .01). The main effects of group for caudal ACC rsFC to a) superior frontal regions, b) left middle frontal regions, and c) medial/dorsal thalamus remained significant (*p* < .01). The group effect for (d) dorsal anterior cingulate to left middle frontal regions was retained (*p* < .02). The group effect for dorsal anterior cingulate to right middle frontal gyrus was reduced to a trend level (*p* = .08). Thus, all but one significant group-based finding were retained when IQ and comorbid substance use (alcohol and nicotine) were covaried within the same analysis.

## Discussion

Chronic cannabis use is increasingly perceived as harmless among US youth. Building upon our group’s prior findings in treated adolescents with CUD (19), this longitudinal study investigated whether chronic cannabis use has the potential for persistent long-range impacts on brain intrinsic functional organization during periods of active neural change in non-treatment-seeking young adults. Results supported three of our hypotheses: a) lack of rsFC increases across time in CUD (observed in HC) between brain regions that mediate sensorimotor processing and attention regulation, b) sustained rsFC alterations in CUD versus HC characterized by lower rsFC between brain regions that mediate executive control and internal awareness, and c) evidence of marginal associations between lower rsFC and binge patterns of cannabis use. Data from the current study did not support our hypothesis of NAcc rsFC alterations in the CUD sample when compared to HC. This study adds compelling longitudinal evidence to the existing literature highlighting *disrupted normative changes* observed in healthy controls and *sustained* disruptions of resting-state networks in young adults with heavy chronic cannabis use. Current findings were limited to examining executive control and reward networks seeded in ACC and NAcc, respectively. Future studies with larger sample sizes and enough power are needed to conduct exploratory analyses examining rsFC of other networks beyond those within the scope of the current study.

### Differences in rsFC Changes Between Groups

Because participants were recruited from a college community within narrow age ranges (∼18–19 years old), all should have experienced similar rsFC changes characteristic of the adolescent-to-early adulthood transition. The lack of a normative increase in caudal ACC rsFC across time in the CUD group (vs. controls) suggests the possibility of long-term effects of cannabis use on brain functional organization. We previously reported similar findings in a different sample of adolescents where a different sample of HCs showed a significant increase in rsFC between caudal ACC and frontal regions across time, while adolescents with CUD did not (19). Current findings indicate that these seemingly detrimental effects extend into early adulthood in an independent sample with CUD who have not sought treatment. This significant interaction was found when examining rsFC of the caudal ACC. There is evidence that this region, known to mediate control of basic functions such as motor control and attention to action such as stimulus–response selection and inhibitory control (48, 49), develops earlier than other ACC regions and continues to develop into early adulthood (17, 50). Those with CUD failed to show the typical increases in intrinsic connectivity of a network needed for motor control seeded in caudal ACC found in HC (17, 19). The implications of this finding need to be further explored with larger samples with more follow-up time points and within the context of behavioral/motor performance.

We have also reported disrupted change in structural connectivity in a subset of these same participants (63). All of the CUD participants in that study are included in the current analysis, with partial overlap of the control sample. Diffusion-weighted data showed lack of axonal fiber organization change in CUD vs. controls between parietal (e.g., superior longitudinal fasciculus) and frontal (e.g., genu of the corpus callosum and white matter adjacent to superior frontal gyrus) regions. These structural alterations are adjacent to, and complement, the current rsFC findings. Cross-sectional evidence has also showed frontal and parietal functional alterations in adolescents and young adults with CUD (51).

### Sustained Group rsFC Differences

While another study has reported immediate detrimental effects of cannabis manifested as reductions in rsFC within the default mode network (52), the current study provides additional supportive evidence of persistent frontal rsFC disruptions (caudal and dorsal ACC) in young adults with CUD. These disruptions could reflect pre-existing liabilities, or it may be that neurotoxic effects of cannabis asymptote persist into young adulthood. Our previous longitudinal fMRI study in adolescents reported that at baseline, rsFC of frontal networks was similar between adolescents with CUD and healthy controls. However, at follow-up, rsFC in individuals with CUD had significantly dropped when compared to HCs (19), supporting the idea that chronic CUD exerts protracted neurotoxic influences on the intrinsic functional organization of networks that mediate executive control, consistent with previous task-based fMRI studies (53). Since rsFC represents the functional architecture of brain networks (54), the quality of rsFC is a measurable manifestation of ongoing state-based brain functional organization that may underlie behavioral alterations (55, 56). Persistent rsFC alterations of motor and cognitive control networks found in CUD may render them vulnerable to motor disinhibition (i.e., caudal ACC) (57), poor conflict monitoring, poor decision making, and poor learning (i.e., dorsal ACC) (29, 58) on a daily basis, which may facilitate further substance misuse.

### Correlations Between Cannabis use Metrics and rsFC

We predicted that lower rsFC strength would be observed in those who used greater amounts of cannabis and with increased frequencies, though we acknowledge that others have failed to find such associations (59, 60). While significant effects in the current study were modest, ostensibly because of the small CUD sample, our findings suggest the possibility of a detrimental impact of binge cannabis use on rsFC.

It should be noted that observed effects of binge cannabis use for the prior 12 months on rsFC remained after controlling for alcohol and nicotine use and that neither alcohol use nor nicotine use was a significant predictor of rsFC alterations in our sample. In this study, binge use was defined by asking participants to report the largest number of hits (deep inhales) consumed in a 24-h period and then to indicate the frequency of use at this level over the past year prior to assessment. Thus, this index reflects use in high amounts and with high frequencies. Greater frequencies of binge cannabis use were marginally associated with decreased rsFC between the dorsal ACC and right middle frontal gyrus, perhaps reflecting loss of control over use behavior, and were nominally associated with lower rsFC in other cingulate-to-frontal connections. Significant correlations with cannabis use have been found with other aspects of neuronal integrity such as correlations with brain structure (e.g., volume) (61) and with task-evoked (N-back task) brain activity (62). Similar to this pattern of findings, the degree of structural (i.e., white matter) changes of frontal and parietal networks was found in our prior report to be positively correlated with the amount of CUD in this sample (63). Counterintuitively, we also observed in the current analysis that a *later* age of CUD onset was associated with decreased rsFC between the dorsal ACC and middle frontal gyrus. While severity of recent cannabis use showed negative associations with dorsal ACC–middle frontal gyrus rsFC (higher recent use correlated with lower rsFC), age of onset of CUD showed a different correlation pattern (later onset of CUD correlated with lower rsFC). This pattern (if replicable) suggests a destabilization of network organization supporting cognitive control known to mediate reward-based decision making and learning (28) earlier versus later in the course of heavy cannabis use. More work is needed to better understand how intrinsic resting-state fluctuations relate to addiction-related symptoms and outcomes. Longitudinal studies that include more than two time points, extend into later adulthood, and have larger samples would permit associations among chronic cannabis exposure, disruptions in neural maturation, and long-term behavioral outcomes to be examined. This study indicates that rsFC of caudal and dorsal anterior cingulate regions should be a focus of continued assessment in CUD.

### Limitations and Future Directions

The following limitations should be addressed in future studies. First, while there was no physiological validation to confirm whether participants remained awake during resting-state scans, it is unlikely they fell asleep, because the average scan time was in the morning (most alert time of day), the resting-state scan was short (37), and participants were instructed to remain awake and were asked if they fell asleep during the scan. A second limitation is that because our sample size is small, the hypothesis-driven analyses examining rsFC of executive control (i.e., ACC) and reward (NAcc) networks need to be confirmed with larger sample sizes suitable for data-driven exploratory analyses of other rsFC networks beyond those within the scope of the current study. Third, because this data set was collected between 2007 and 2013, our imaging data resolution (TR = 2 s) is not optimal for more advanced data processing and analyses utilizing the latest processing pipelines (64). Fourth, the ability to control for premorbid characteristics is notoriously difficult in studies of active substance users. Our design does not permit causal interpretations given that the CUD sample was not studied prior to use onset. The sustained rsFC alterations observed in CUD could predate use onset (65). Fifth, while participants with CUD were asked to remain abstinent from cannabis, alcohol, and other substances including nicotine for 24 h prior to each scanning session, we did not confirm abstinence with formal drug testing. Thus, there is a possibility that THC or alcohol levels were higher in the CUD group, potentially influencing findings. Additionally, while our analyses did control for reported amounts of cigarette use, there is a slight possibility that current findings are related to craving for nicotine. However, we maintain that nicotine craving is unlikely because i) participants were not daily cigarette smokers and ii) the patterns of activation and group differences that we observed are not consistent with the craving literature. Specifically, there is evidence that nicotine craving involves nucleus accumbens–orbitofrontal resting functional connectivity (66), a network that was not found to have significant effects in the current study (i.e., from our NAcc rsFC analyses). Future studies collecting THC and alcohol levels immediately before the scanning sessions and the inclusion of measures of craving (for both cannabis and nicotine) should be conducted. Sixth, our unanticipated results showing a correlation between later onset of CUD and lower rsFC should be replicated with a larger sample characterized by greater representation of earlier ages of use onset so that these findings can be replicated and confirmed. Finally, to be able to model a developmental trajectory of the effects of cannabis on brain functional organization, larger-scale longitudinal studies that examine rsFC with more than two time points from childhood and early adolescence into adulthood would provide more nuanced depictions of dynamic changes and disruptions as use progresses from initiation into chronic cannabis use during development (67).

### Conclusions

Intrinsic functional organization of the brain continues to change in early adulthood. The current study provides evidence that these changes may be altered in the context of chronic cannabis use. There are relatively few studies that have assessed intrinsic functional connectivity in the context of heavy cannabis use. We observed disruption in the circuitry of the anterior cingulate cortex that underlies sensorimotor and cognitive control in young adults with CUD, which could not be attributed to comorbid nicotine and alcohol use and which may have longer-range impacts on behavior. Reproducibility of the current findings using more advanced neuroimaging collection parameters (e.g., Human Connectome Project, Adolescent Brain Cognitive Development Study) that allow the use of more updated processing pipelines (61) with higher spatial and temporal resolution is needed. Future work should focus on prospective investigations to help disentangle dose-dependent from pre-existing effects. The current findings indicate that ACC connectivity and its associations with behavior should be a focus in such investigations.

## Data Availability

The datasets generated for this study are available on request to the corresponding author.

## Ethics Statement

This study was carried out in accordance with the recommendations of the University of Minnesota IRB Committee with written informed consent from all subjects. All subjects gave written informed consent in accordance with the Declaration of Helsinki. The protocol was approved by the University of Minnesota IRB Committee.

## Author Contributions

ML and PC were responsible for the study concept and design. MB, PC, and ML contributed to the acquisition of data. JC, ML, PC, MB, and KL contributed to data analysis and interpretation of findings. JC, ML, and PC drafted the manuscript. JC, ML, and PC provided critical revisions of the manuscript for important intellectual content. All authors critically reviewed content and approved the final version for publication. The authors have no conflicts of interest to declare.

## Funding

Data collection and analysis was supported by the National Institute on Drug Abuse [R01DA017843 (to ML)] and the National Institute on Alcohol Abuse and Alcoholism grants [AA020033 (to ML)] and by grants to the University of Minnesota’s Center for Magnetic Resonance Research (BTRC P41 EB015894, P30 NS076408). Analyses were also supported by funding from the National Institute of Health [KL2TR002492 (to JC)], the National Institute on Alcohol Abuse and Alcoholism [K01AA026349-01A1 (to JC)], and the National Institute on Drug Abuse [R01DA038984 (to KL)]. The content is solely the responsibility of the authors and does not necessarily represent the official views of the National Institutes of Health.

## Conflict of Interest Statement

The authors declare that the research was conducted in the absence of any commercial or financial relationships that could be construed as a potential conflict of interest.

## Abbreviations

ACC, anterior cingulate cortex; AFNI, Analysis of Functional NeuroImages; CUD, cannabis use disorder; *DSM-IV*, *Diagnostic and Statistical Manual of Mental Disorders-IV;* fMRI, functional magnetic resonance imaging; HC, healthy controls; K-SADS, Kiddie Schedule for Affective Disorders and Schizophrenia; MELODIC, Multivariate Exploratory Linear Optimized Decomposition into Independent Components; MNI, Montreal Neurological Institute; NAcc, nucleus accumbens; PEI, Personal Experience Inventory; rsFC, resting-state functional connectivity

## Footnotes

1 The longitudinal study from which the controls were derived was fully funded for two time points; the third time point was not initially budgeted and was only partially completed before funding lapsed. A new round of funding supported the fourth assessment wave.

2
https://afni.nimh.nih.gov/pub/dist/doc/program_help/3dvolreg.html

3
http://fsl.fmrib.ox.ac.uk/fsl/fslwiki/MELODIC

4
http://www.fmrib.ox.ac.uk/fslcourse/lectures/melodic.pdf

5
https://afni.nimh.nih.gov/pub/dist/doc/program_help/3dROIstats.html

6
https://afni.nimh.nih.gov/pub/dist/doc/program_help/3dfim+.html

7
https://afni.nimh.nih.gov/pub/dist/doc/program_help/3dcalc.html

8
https://afni.nimh.nih.gov/pub/dist/doc/program_help/3dLME.html

9
https://afni.nimh.nih.gov/pub/dist/doc/program_help/3dFWHMx.html

10
https://afni.nimh.nih.gov/pub/dist/doc/program_help/3dClustSim.html

11
https://afni.nimh.nih.gov/pub/dist/doc/program_help/3dmerge.html
